# Therapeutic Potential of Astrocyte-Derived Extracellular Vesicles in Post-Stroke Recovery: Behavioral and MRI-Based Insights from a Rat Model

**DOI:** 10.3390/life15091418

**Published:** 2025-09-09

**Authors:** Yessica Heras-Romero, Axayácatl Morales-Guadarrama, Luis B. Tovar-y-Romo, Diana Osorio Londoño, Roberto Olayo-González, Ernesto Roldan-Valadez

**Affiliations:** 1 Physics Department, Universidad Autónoma Metropolitana, Iztapalapa, Mexico City 09340, Mexico; 2 Electrical Engineering Department, Universidad Autónoma Metropolitana, Iztapalapa, Mexico City 09340, Mexico; amorales@ci3m.mx (A.M.-G.); dmol@xanum.uam.mx (D.O.L.); 3 Medical Imaging and Instrumentation Research National Center, Universidad Autónoma Metropolitana, Iztapalapa, Mexico City 09340, Mexico; 4 Department of Molecular Neuropathology, Instituto de Fisiología Celular, Universidad Nacional Autónoma de México, Mexico City 04510, Mexico; ltovar@ifc.unam.mx; 5 Division of Neurosciences, Instituto Nacional de Rehabilitación ‘Luis Guillermo Ibarra Ibarra’, Mexico City 14389, Mexico; 6 Department of Radiology, I.M. Sechenov First Moscow State Medical University (Sechenov University), 119992 Moscow, Russia

**Keywords:** astrocyte-derived extracellular vesicles, ischemic stroke, diffusion tensor imaging, neuroregeneration, MRI biomarkers, rat model

## Abstract

Astrocyte-derived extracellular vesicles (ADEVs) have emerged as promising neuroprotective agents for ischemic stroke. In this study, we evaluated the therapeutic potential of hypoxia-conditioned ADEVs (HxEVs) administered intracerebroventricularly in a rat model of transient middle cerebral artery occlusion (tMCAO). Serial magnetic resonance imaging (MRI) with diffusion tensor imaging (DTI) was performed at 1, 7, 14, and 21 days post-stroke. HxEV treatment produced a significant reduction in infarct volume from day 1, sustained through day 21, and was accompanied by improvements in motor and sensory recovery. DTI analyses showed progressive normalization of fractional anisotropy (FA) and radial diffusivity (RD), particularly in the corpus callosum and striatum, reflecting microstructural repair. In contrast, mean diffusivity (MD) was less sensitive to these treatment effects. Regional differences in therapeutic response were evident, with earlier and more sustained recovery in the corpus callosum than in other brain regions. Histological findings confirmed greater preservation of dendrites and axons in HxEV-treated animals, supporting the role of these vesicles in accelerating post-stroke neurorepair. Together, these results demonstrate that hypoxia-conditioned ADEVs promote both structural and functional recovery after ischemic stroke. They also highlight the value of DTI-derived biomarkers as non-invasive tools to monitor neurorepair. The identification of region-specific therapeutic effects and the validation of reliable imaging markers provide a strong foundation for future research and development.

## 1. Introduction

Ischemic stroke remains a leading cause of adult disability and death worldwide. Despite advances in acute reperfusion therapies such as thrombolysis and mechanical thrombectomy, effective strategies to promote neural repair and functional recovery during the subacute and chronic phases are limited [1]. This therapeutic gap has driven interest in cell-based approaches and their derivatives—particularly extracellular vesicles (EVs)—as candidates for neurorestorative interventions.

Astrocytes, the most abundant glial cells in the central nervous system (CNS), are essential for neural homeostasis and the response to injury. After cerebral ischemia they become reactive and participate in inflammation, repair, and tissue remodeling [2]. One emerging mechanism by which astrocytes modulate the post-stroke milieu is the release of EVs—membrane-bound particles carrying proteins, lipids, and nucleic acids that influence recipient cells and show therapeutic promise in preclinical models [3,4].

Astrocyte-derived EVs (ADEVs) are of particular interest because of their neuroprotective and neuroregenerative properties. Prior studies show that ADEVs can modulate inflammation, reduce apoptosis, and promote angiogenesis and synaptic plasticity in CNS injury models [5,6,7]. EVs from mesenchymal stem cells similarly reduce infarct volume and enhance functional recovery in rodent stroke models, and EVs can be engineered to deliver therapeutic molecules (e.g., microRNAs), further expanding their potential for brain repair [6].

Beyond therapy, EVs hold promise as biomarkers: their detection in blood and cerebrospinal fluid enables monitoring of disease progression and treatment response [7]. For example, higher concentrations of aquaporin-4 and glial-cell-line-derived neurotrophic factor (GDNF) in astrocyte-derived EVs have been correlated with stroke severity and clinical outcome [3].

Advanced neuroimaging, particularly magnetic resonance imaging (MRI), has likewise facilitated biomarker discovery in CNS injury. Structural MRI and diffusion tensor imaging (DTI) provide insight into white-matter integrity and tract disruption, improving prognostication and rehabilitation planning. Recent work indicates that DTI-derived metrics can reveal microstructural patterns associated with tissue viability and damage, aiding more accurate assessment of lesion severity and recovery potential [8].

Against this background, the present study evaluated the therapeutic efficacy of intracerebroventricularly administered ADEVs in a rat model of ischemic stroke induced by transient middle cerebral artery occlusion (tMCAO). We combined behavioral assessments with MRI to characterize structural and microstructural changes associated with ADEV treatment. These findings add to the growing evidence supporting ADEVs as agents to enhance post-stroke recovery and clarify their interaction with imaging biomarkers in stroke therapeutics.

## 2. Materials and Methods

### 2.1. Animals

Male Wistar rats (6 weeks old; 270–280 g) were obtained from the Animal Facility of the Instituto de Fisiología Celular (IFC), Universidad Nacional Autónoma de México (UNAM). All procedures complied with the Mexican standard NOM-062-ZOO-1992 and were approved by the Scientific Committee (protocol code CI3M08-22, approved on 5 December 2022). The study was designed and reported in accordance with ARRIVE 2.0 guidelines [9]. Animals were housed individually in temperature-controlled rooms under a 12:12 h light/dark cycle with ad libitum access to food and water.

### 2.2. Experimental Design

Animals were randomly assigned to three groups (*n* = 6 per group) [10,11]:

(1) Intact controls;
(2) tMCAO plus intracerebroventricular (i.c.v.) injection of vehicle (0.9% NaCl);
(3) tMCAO plus i.c.v. astrocyte-derived extracellular vesicles obtained under hypoxic conditions (HxEVs).

Sample size was determined from a pilot study and a priori power analysis (Cohen’s d > 0.3, β = 0.8, α = 0.05), accounting for an anticipated post-surgical mortality of 20%. MRI and behavioral assessments were performed at 1, 7, 14, and 21 days post-tMCAO. Inclusion criteria were: ≥50% reduction in cerebral perfusion during 60 min occlusion (confirmed by laser Doppler), absence of hemorrhage, and survival to day 21. Humane endpoints were predefined for animals exhibiting hemiplegia or severe weakness that impaired feeding or drinking. A separate cohort (*n* = 4 per group) was used for immunohistochemistry at day 21. Only male rats were studied to avoid the estrogen-related neuroprotection reported in females [12].

### 2.3. MCAO Stroke Model

Rats were anesthetized with isoflurane (5% for induction; 1.5–2% for maintenance) delivered in 100% oxygen at 400 mL/min. Core temperature was maintained at 37.7 ± 0.5 °C using a feedback-controlled heating pad. Cerebral blood flow (CBF) in the MCA territory was monitored by laser Doppler flowmetry (Perimed, probe model 407) at stereotactic coordinates AP −2.0 mm, L −3.5 mm relative to bregma; signals were sampled every 0.3 s with PeriSoft software.

The left common carotid artery (CCA) and its branches were exposed with care to preserve the vagus nerve. The distal external carotid artery (ECA) was ligated with 6-0 silk, and microvascular clips were placed on the internal carotid artery (ICA) and proximal CCA. A 7-0 silicone-coated nylon monofilament (DOCCOL-403734) was introduced through an arteriotomy in the ECA and advanced into the ICA until resistance was encountered at the MCA origin. Successful occlusion was defined as a ≥65% reduction in CBF. After 60 min of occlusion, the filament was withdrawn to allow reperfusion.

This procedure enables the establishment of a reliable in vivo model of cerebral hypoxia, as the transient occlusion of the middle cerebral artery significantly reduces cerebral blood flow for 60 min, inducing a sustained hypoxic condition. Subsequent reperfusion simulates ischemia–reperfusion injury, closely replicating the pathophysiology of ischemic stroke.

### 2.4. Postoperative Care

After surgery, animals were maintained on a heated pad until fully recovered. Buprenorphine (0.05 mg/kg, s.c.) was administered for analgesia [10], and 1 mL saline (i.p.) was given to prevent dehydration.

### 2.5. Primary Astrocyte Cultures and EV Isolation

Primary astrocytes were prepared from the cortices of 1–2-day-old Wistar rats following established protocols [11]. Tissue was minced, digested with trypsin (1 mg/mL, 10 min, room temperature), and mechanically dissociated. The cell suspension was centrifuged and resuspended in DMEM/F12 supplemented with 10% fetal bovine serum (FBS; Gibco, Thermo Fisher Scientific, Waltham, MA, USA ). Cells were plated in poly-D-lysine–coated flasks. After 24 h, cultures were shaken overnight (200 rpm) to remove non-adherent cells. Cultures (>98% GFAP+) were used between passages 3–5.

For EV isolation, astrocytes were exposed to hypoxia (100% N_2_, 6 h, 37 °C) and then returned to normoxia for 42 h. Conditioned medium was sequentially filtered (0.22 μm/220 nm), centrifuged at 50,000× *g* for 30 min to remove debris, and ultracentrifuged at 100,000× *g* for 70 min to pellet EVs. This protocol provides a consistent in vitro hypoxia model that simulates the oxygen-deprived milieu of ischemic stroke.

### 2.6. Exosome Characterization and Administration

For transmission electron microscopy (TEM), EV suspensions were loaded onto glow-discharged 400-mesh copper grids, stained with 2% uranyl formate, and imaged on a JEOL JEM-1200 microscope (80 keV). Protein concentration was determined by the Lowry assay.

Hypoxia-conditioned astrocyte-derived EVs (HxEVs; 400 ng protein in 4 μL PBS) were administered intracerebroventricularly (i.c.v.) 30 min after reperfusion using a stereotaxic microcapillary (<50 μm tip) into the contralateral ventricle (coordinates: AP −0.8 mm, ML −1.5 mm, DV −4.0 mm from bregma).

### 2.7. Behavioral Assessments

Sensorimotor function was evaluated with a validated 28-point neurological deficit scale at 1, 7, 14, and 21 days post-stroke (0 = severe deficit; 28 = normal). All tests were video-recorded and scored by blinded observers [12,13].

### 2.8. Magnetic Resonance Imaging

MRI was performed at 1, 7, 14, and 21 days post-MCAO on a 3T Philips Achieva scanner with a 16-channel neurovascular coil. Anesthesia consisted of xylazine (7 mg/kg) and 2% isoflurane [14]. The sequences acquired were

1. T1-weighted (3D TFE): TE 4.6 ms, TR 9.9 ms, voxel 0.8 × 0.8 × 0.8 mm, 140 slices.
2. T2-weighted (SE): TE 250 ms, TR 2500 ms, voxel 0.9 × 0.9 × 0.9 mm, 346 slices.
3. DTI: 32 directions, b = 800 s/mm^2^, TE 84 ms, TR 2521 ms, voxel 1.51 × 1.54 × 1.5 mm.

DTI data were processed in DSI Studio and DTI Studio. Eigenvalues (λ1, λ2, λ3) were used to generate maps of fractional anisotropy (FA), mean diffusivity (MD), axial diffusivity (AD), and radial diffusivity (RD). Microstructural integrity was assessed in the cortex, striatum, and corpus callosum following Cortez-Conradis et al. [15].

### 2.9. Assessment of Infarct Volume and Neurological Function

Infarct volume was quantified at 24 h and at 7, 14, and 21 days post-MCAO from T2/FLAIR images. Lesions were manually segmented in AMIRA, and volumetry was performed in DSI Studio by multiplying segmented voxels by voxel size; results are reported in mm^3^. Neurological function was evaluated with a composite score adapted from the modified neurological severity score (mNSS), encompassing motor activity, sensory responses, reflexes, and balance. Testing was performed at baseline and at 1, 7, 14, and 21 days to track recovery trajectories. Forelimb asymmetry (cylinder test) and sensorimotor coordination (adhesive removal test) were also assessed. All evaluations were performed by observers blinded to group allocation.

### 2.10. Immunofluorescence and Confocal Microscopy

At day 21, animals were deeply anesthetized with pentobarbital (100 mg/kg) and perfused transcardially with 0.9% NaCl followed by 4% paraformaldehyde (PFA). Brains were post-fixed in 4% PFA for 24 h, cryoprotected in 30% sucrose, and sectioned at 40 μm. Sections were permeabilized with 0.5% Triton X-100, blocked in 5% bovine serum albumin, and incubated for 48 h at 4 °C with anti-MAP2 (1:200, Invitrogen PAS-17646) and DAPI (1:200, Millipore). After washing, sections were incubated for 2 h at room temperature with Alexa Fluor 647-conjugated secondary antibody (1:300, ThermoFisher A32723). Z-stacks (~45 optical sections at 0.5 μm intervals) were acquired on a Zeiss LSM 800 confocal microscope using a 20× objective.

### 2.11. Summary of Experimental Strategy

**Figure 1:**
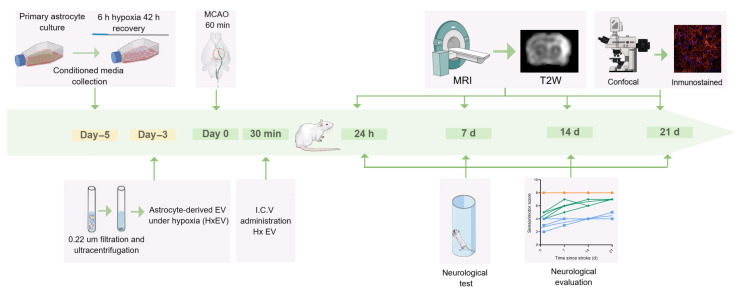
Experimental timeline. In rats subjected to transient middle cerebral artery occlusion (tMCAO), hypoxia-conditioned astrocyte-derived extracellular vesicles (HxEVs) were administered by stereotaxic intracerebroventricular (i.c.v.) injection into the hemisphere contralateral to the infarct 30 min after the onset of reperfusion to evaluate effects on infarct evolution. Intact (orange), Vehicle (blue), and HxEV (green).

HxEVs isolated from hypoxia-conditioned astrocyte cultures were stereotaxically injected i.c.v. into the hemisphere contralateral to infarction 30 min after reperfusion in MCAO-subjected rats to evaluate effects on stroke progression (Figure 1).

### 2.12. Statistical Analysis

Analyses were performed in GraphPad Prism v9.0.2 (GraphPad Software, San Diego, CA, USA). Data normality was assessed with the Shapiro–Wilk test; because distributions deviated from normality, non-parametric methods were used.

Between-group comparisons (Intact, Vehicle, HxEV) of diffusion MRI metrics—fractional anisotropy (FA), axial diffusivity (AD), radial diffusivity (RD), and mean diffusivity (MD)—within the cortex, striatum, and corpus callosum were evaluated with the Kruskal–Wallis test followed by Dunn’s post hoc multiple-comparisons test. Longitudinal within-group changes across days 1, 7, 14, and 21 were analyzed using the Friedman test with Wilcoxon signed-rank tests for pairwise time point contrasts. Direct two-group comparisons (Vehicle vs. HxEV, HxEV vs. Intact, Vehicle vs. Intact) were assessed with the Mann–Whitney U test.

All tests were two-tailed, and *p* < 0.05 was considered statistically significant.

## 3. Results

### 3.1. Astrocyte-Derived EVs Reduce Infarct Volume and Improve Neurofunctional Recovery After Ischemic Stroke

Primary astrocytes were obtained from newborn Wistar rats (P1–P2) following the previously described protocol. Cells were exposed to hypoxia for 6 h and then returned to normoxia for 42 h (HxEV). To ensure that vesicles originated from astrocytes, conditioned medium contained exosome-depleted FBS, and extracellular vesicles were isolated by differential centrifugation.

**Figure 2:**
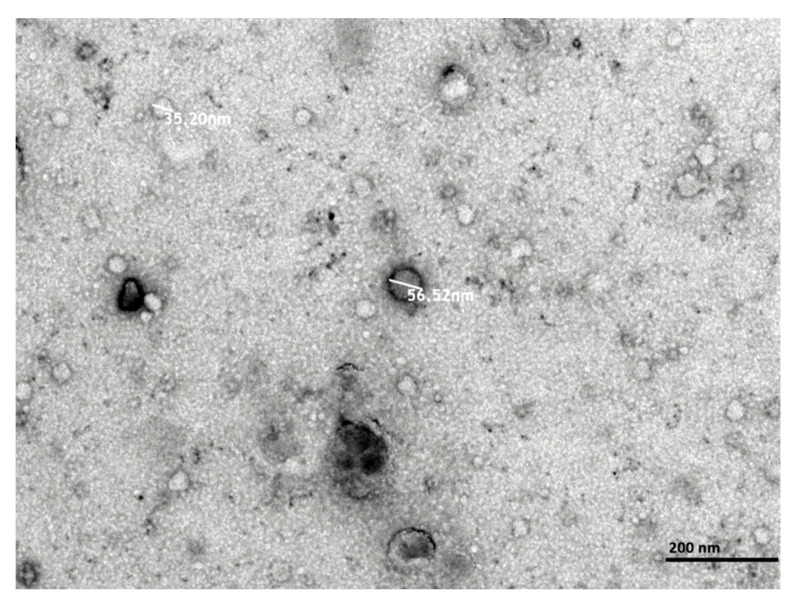
Transmission electron microscopy (TEM) micrographs of hypoxia-conditioned astrocyte-derived extracellular vesicles (HxEVs). Vesicles were visualized by negative staining with uranyl formate on copper/carbon-coated grids. Scale bars: 200 nm.

Transmission electron microscopy (TEM) confirmed a population of small, round vesicles with morphology characteristic of exosomes (Figure 2). Vesicle size ranged from 30 to 150 nm, consistent with the exosome/small-EV fraction according to International Society for Extracellular Vesicles (ISEV) criteria, indicating successful isolation of a homogeneous, well-defined EV preparation.

We then evaluated the in vivo impact of these hypoxia-conditioned astrocyte-derived EVs on infarct evolution and neurofunctional recovery in the rat ischemic stroke model.

Representative coronal MRI from Vehicle- and HxEV-treated rats at 1, 7, 14, and 21 days post-stroke. Left: native T2-weighted FSEMS images. Right: pseudocolor overlays highlight infarcted tissue. Early damage involves the striatum with partial cortical extension; infarcted regions diminish over time in HxEV-treated animals. (

To assess the effects of hypoxia-conditioned astrocyte-derived extracellular vesicles (HxEVs) after tMCAO, we quantified infarct volume by T2-weighted fast spin echo multislice (FSEMS) MRI and evaluated neurological deficits with standardized behavioral tests.

**Figure 3:**
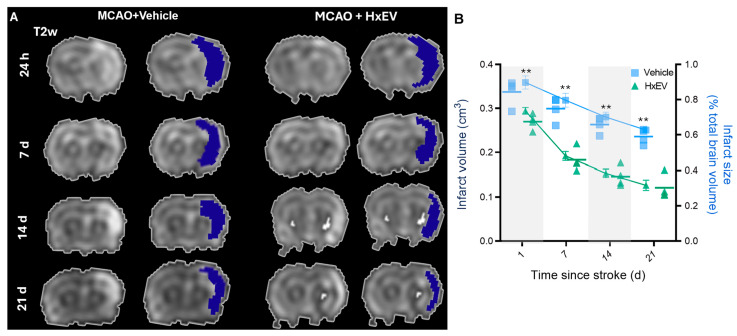
Intracerebroventricular HxEVs reduce infarct burden after tMCAO. (**A**) Representative coronal MRI from Vehicle- and HxEV-treated rats at 1, 7, 14, and 21 days post-stroke. Left: native T2-weighted FSEMS images. Right: pseudocolor overlays highlight infarcted tissue. Early damage involves the striatum with partial cortical extension; infarcted regions diminish over time in HxEV-treated animals. (**B**) Quantification of infarct volume (left *y*-axis, dark blue) and percent infarct relative to total brain volume (right *y*-axis, light blue) at each time point. Data are mean ± SEM (*n* = 6 per group). Vehicle vs. HxEV comparisons at each time point were analyzed with Mann–Whitney U tests; ** *p* ≤ 0.01.

At 24 h post-stroke, MRI showed extensive ischemic damage localized primarily to the striatal core with partial involvement of the primary motor and somatosensory cortices, a pattern consistent with MCA-territory infarction (Figure 3A). Across subsequent time points, rats receiving intracerebroventricular (i.c.v.) HxEVs exhibited visibly smaller lesions than Vehicle-treated controls (Figure 3B). Quantitative analysis confirmed a significant reduction in infarct volume in the HxEV group as early as day 1 (*p* = 0.002), with sustained and greater reductions at days 14 and 21 (*p* = 0.005). These findings indicate that early i.c.v. administration of HxEVs confers durable neuroprotection against ischemic injury.

**Figure 4:**
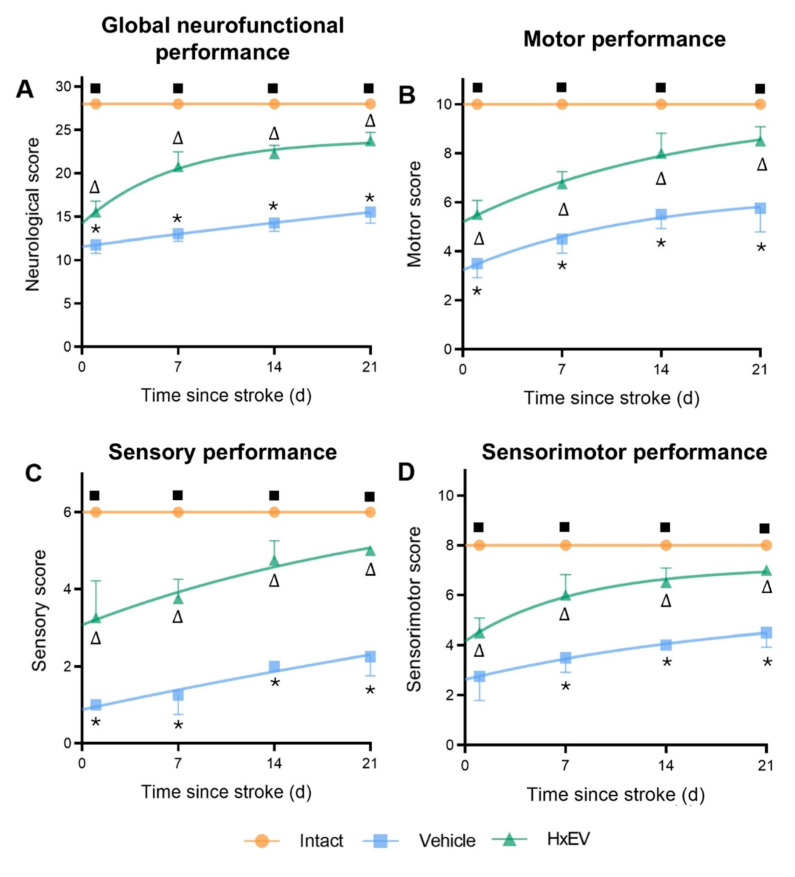
Astrocyte-derived extracellular vesicles (HxEVs) enhance neurofunctional recovery after ischemic stroke. Mean ± SEM neurofunctional scores are shown for each group at days 1, 7, 14, and 21 post-stroke (*n* = 6 per group). Panels: (**A**) global neurological score; (**B**) motor performance; (**C**) sensory performance; (**D**) integrated sensorimotor score. Between-group differences at each time point were assessed with Mann–Whitney U tests. Symbols denote pairwise significance: * Vehicle vs. HxEV; ∆ HxEV vs. Intact; ∎ Intact vs. Vehicle (all *p* ≤ 0.05). Higher scores indicate better function; HxEV-treated rats exhibited significantly greater recovery than Vehicle-treated controls across all domains.

Neurofunctional performance paralleled the structural findings. Using the composite modified neurological severity score (mNSS; Table A1, Appendix A), HxEV-treated rats showed significantly better recovery than Vehicle controls (Figure 4), with differences evident by day 7 and sustained through day 21.

- Total neurological score (Figure 4A): HxEV-treated rats scored consistently higher from day 1 onward (*p* < 0.05).
- Motor subscore (Figure 4B): Recovery was faster and more pronounced in the HxEV group beginning on day 1 (*p* < 0.05).
- Sensory subscore (Figure 4C): Treated animals showed progressive sensory improvement versus Vehicle (*p* < 0.05).
- Sensorimotor score (Figure 4D): HxEVs improved integrative sensorimotor function, with significant differences from Vehicle by day 7 and persisting through day 21 (*p* < 0.05).

Collectively, these structural and behavioral data indicate that i.c.v. HxEV administration shortly after reperfusion confers time-dependent neuroprotection and accelerates neurological recovery after ischemic stroke.

### 3.2. Diffusion Tensor Imaging Reveals Microstructural Restoration Mediated by HxEVs

To further examine post-stroke recovery, we analyzed diffusion tensor imaging (DTI) biomarkers—axial diffusivity (AD), radial diffusivity (RD), mean diffusivity (MD), and fractional anisotropy (FA)—in cortex, striatum, and corpus callosum over 21 days.

**Figure 5:**
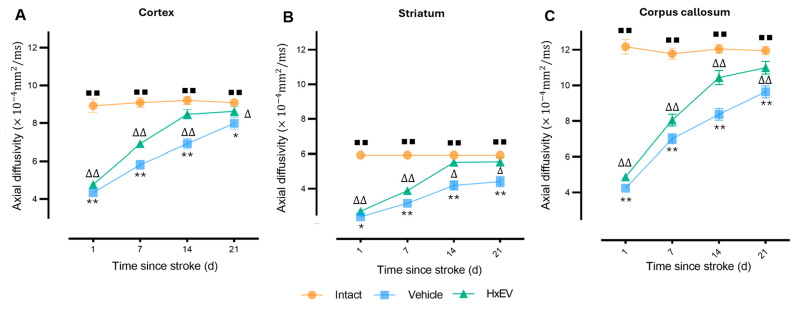
Longitudinal axial diffusivity (AD) changes in cortex, striatum, and corpus callosum. AD trajectories are shown for Intact (orange), Vehicle (blue), and HxEV (green) groups in (**A**) cortex, (**B**) striatum, and (**C**) corpus callosum. Within-group longitudinal effects were evaluated with the Friedman test. Pairwise group comparisons at each time point were performed with Mann–Whitney U tests (*p* < 0.05). Symbols denote significance: */** Vehicle vs. HxEV (*p* ≤ 0.05/*p* ≤ 0.01); ∆/∆∆ HxEV vs. Intact (*p* ≤ 0.05/*p* ≤ 0.01); ∎∎ Intact vs. Vehicle (*p* ≤ 0.01).

Axial diffusivity (AD). AD fell sharply at day 1 in cortex and striatum, consistent with acute axonal injury (Figure 5A,B). In HxEV-treated rats, AD progressively normalized from day 7 onward (*p* < 0.05), with significant recovery evident in the striatum by day 14 (*p* < 0.05), whereas Vehicle animals showed minimal improvement. In the corpus callosum (Figure 5C)—a major white-matter tract prone to ischemic demyelination—HxEV administration produced sustained AD enhancement from day 1 (*p* < 0.01), indicating robust axonal preservation.

**Figure 6:**
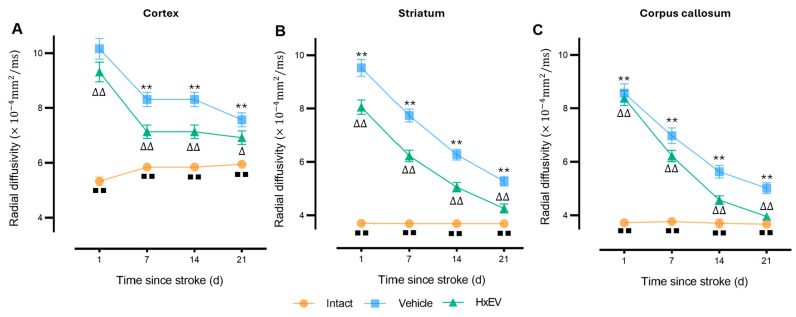
Longitudinal radial diffusivity (RD) in cortex, striatum, and corpus callosum. RD trajectories (mean ± SEM; *n* = 6 per group) are shown for Intact (orange), Vehicle (blue), and HxEV (green) in (**A**) cortex, (**B**) striatum, and (**C**) corpus callosum. Within-group longitudinal effects were tested with the Friedman test. Between-group comparisons at each time point used Mann–Whitney U tests. Except for HxEV vs. Vehicle on day 1 in the cortex (*p* = 0.42), all pairwise contrasts were significant. Symbols denote significance: ** Vehicle vs. HxEV (*p* ≤ 0.01); ∆/∆∆ HxEV vs. Intact (*p* ≤ 0.05/*p* ≤ 0.01); ∎∎ Intact vs. Vehicle (*p* ≤ 0.01).

Radial diffusivity (RD)—a marker of demyelination—was significantly elevated in all regions at day 1 post-stroke. From day 7 onward, HxEV-treated rats showed a progressive decline in RD (*p* < 0.005), most prominently in the corpus callosum (Figure 6). By day 21, RD in the HxEV group approached Intact levels (*p* ≤ 0.01), whereas Vehicle animals remained elevated, consistent with persistent white-matter disruption.

**Figure 7:**
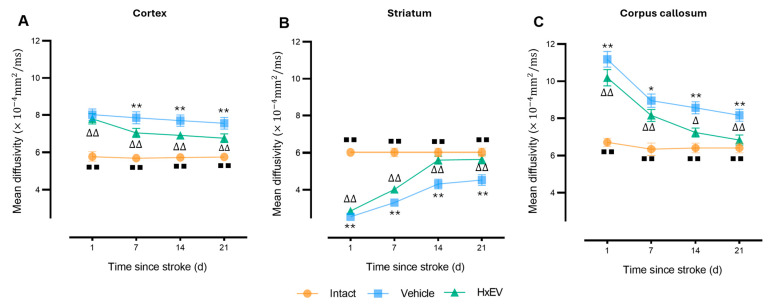
Longitudinal mean diffusivity (MD) in cortex, striatum, and corpus callosum. MD trajectories (mean ± SEM; *n* = 6 per group) are shown for Intact (orange), Vehicle (blue), and HxEV (green) in (**A**) cortex, (**B**) striatum, and (**C**) corpus callosum. Within-group longitudinal effects were analyzed with the Friedman test. Pairwise group comparisons at each time point used Mann–Whitney U tests. Except for HxEV vs. Vehicle on day 1 in the cortex (*p* = 0.26), all pairwise contrasts were significant (*p* < 0.05). Symbols denote significance: */** Vehicle vs. HxEV (*p* ≤ 0.05/*p* ≤ 0.01); ∆/∆∆ HxEV vs. Intact (*p* ≤ 0.05/*p* ≤ 0.01); ∎∎ Intact vs. Vehicle (*p* ≤ 0.01).

Mean diffusivity (MD)—a global index of water mobility—was acutely elevated in both tMCAO groups at day 1 (*p* < 0.005), consistent with edema and cytotoxic injury. Thereafter, MD normalized more rapidly in HxEV-treated rats, particularly in cortex and striatum (Figure 7A,B). In contrast, Vehicle-treated animals showed persistently elevated MD, indicating delayed edema resolution and poorer microstructural recovery.

**Figure 8:**
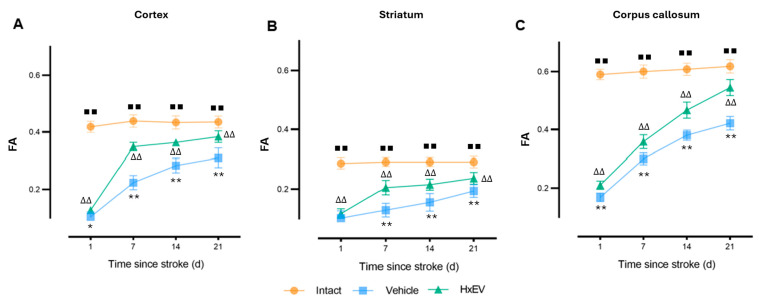
Longitudinal fractional anisotropy (FA) in cortex, striatum, and corpus callosum. FA trajectories (mean ± SEM; *n* = 6 per group) are shown for Intact (orange), Vehicle (blue), and HxEV (green) in (**A**) cortex, (**B**) striatum, and (**C**) corpus callosum. Within-group longitudinal effects were tested with the Friedman test. Pairwise group comparisons at each time point used Mann–Whitney U tests. All pairwise contrasts were significant (*p* < 0.05) except HxEV vs. Vehicle on day 1 in the striatum (*p* = 0.28). Symbols denote significance: */** Vehicle vs. HxEV (*p* ≤ 0.05/*p* ≤ 0.01); ∆∆ HxEV vs. Intact (*p* ≤ 0.01); ∎∎ Intact vs. Vehicle (*p* ≤ 0.01). Higher FA reflects greater microstructural organization.

Fractional anisotropy (FA) emerged as the most sensitive and robust DTI marker. FA fell sharply after stroke across all regions. In HxEV-treated rats, FA recovered significantly from day 7 onward—most prominently in the corpus callosum (Figure 8C; *p* < 0.01)—with improvements also evident in the striatum and motor cortex (Figure 8A,B). In Vehicle controls, FA remained markedly reduced throughout the study, indicating impaired microstructural reorganization.

These data collectively indicate that HxEV treatment facilitates axonal repair, remyelination, and restoration of microstructural integrity in both white and gray matter, as reflected by dynamic changes in DTI biomarkers.

### 3.3. Structural Correlates of Neural Repair and Validation of DTI Findings

To validate the neuroimaging results and assess structural correlates of neural integrity, immunofluorescence was performed on brain sections harvested 21 days after tMCAO. Neuronal and dendritic integrity were evaluated by microtubule-associated protein-2 (MAP2) labeling in peri-infarct cortical and striatal regions, providing histological evidence complementary to the DTI metrics.

Quantitative assessment of dendritic preservation was performed by measuring the MAP2-positive area within predefined regions of interest. HxEV-treated animals exhibited significantly greater MAP2-positive coverage than Vehicle controls (*p* < 0.05).

**Figure 9:**
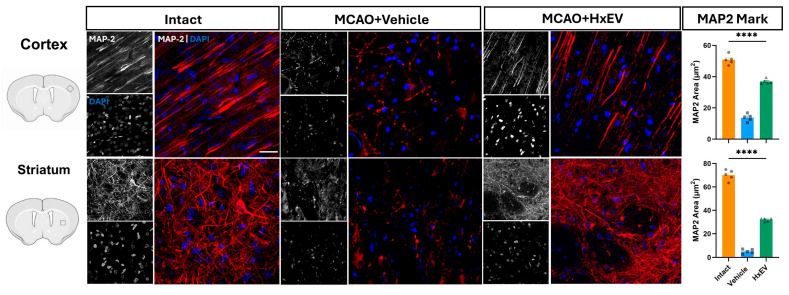
MAP2 immunofluorescence at day 21. Representative confocal Z-projections (10–15 optical sections) from 40-µm sections stained for MAP2 with Alexa Fluor 647 (red) in the ipsilateral motor cortex and dorsal striatum of Intact, Vehicle, and HxEV groups. Scale bars: 50 µm. Right, quantification of MAP2-positive area (“MAP2 covered area”) shown as mean ± SEM with individual data points. Group differences were analyzed by one-way ANOVA with Tukey’s post hoc test; **** *p* < 0.0001 for all pairwise comparisons among Intact, Vehicle, and HxEV.

In Vehicle-treated rats, MAP2 staining revealed marked disruption of neuronal morphology and dendritic architecture. The peri-infarct cortex showed reduced MAP2-positive neuronal density, fragmented dendritic arbors, and shortened processes (Figure 9). These histological alterations were concordant with the imaging findings of reduced FA and elevated RD.

In contrast, rats treated with hypoxia-conditioned astrocyte-derived extracellular vesicles (HxEVs) showed preservation of MAP2-positive neurons and dendritic networks. The peri-infarct cortex exhibited dense, uniform MAP2 immunoreactivity, with apical dendrites displaying clear orientation and continuity. Z-stack confocal imaging further revealed intact dendritic spines and microtubule architecture in this group.

The striatum of HxEV-treated animals likewise demonstrated improved dendritic arborization and preserved neuronal morphology relative to Vehicle controls, consistent with the partial recovery in FA and RD within this region (Figure 9).

**Figure 10:**
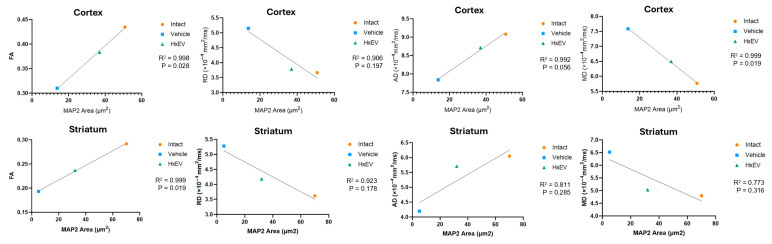
Correlation of DTI metrics with MAP2 immunostaining. Pearson correlation and simple linear regression were used to relate MAP2-positive (covered) area to diffusion tensor metrics—fractional anisotropy (FA), radial diffusivity (RD), axial diffusivity (AD), and mean diffusivity (MD).

To statistically relate histology to imaging, we performed correlation analyses between MAP2-covered area and DTI biomarkers. Pearson correlation and simple linear regression demonstrated overall concordance between modalities, supporting the validity of DTI as a surrogate marker of dendritic integrity (Figure 10).

A strong correlation was observed among structural biomarkers overall (R^2^ ≥ 0.9 between MAP2-covered area and DTI metrics). In the cortex, MAP2 correlated significantly with FA (*p* = 0.02) and MD (*p* = 0.019), but not with RD (*p* = 0.19) or AD (*p* = 0.056), indicating that dendritic integrity is better captured by global diffusion properties than by direction-specific diffusivities. In the striatum, MAP2 correlated significantly with FA (*p* = 0.019), but not with RD (*p* = 0.17), AD (*p* = 0.28), or MD (*p* = 0.31). Although FA values are generally lower in the striatum owing to abundant crossing fibers, FA nevertheless showed a robust association with MAP2.

Together, these results support the validity of DTI-derived metrics as quantitative biomarkers for in vivo microstructural analysis.

**Figure 11:**
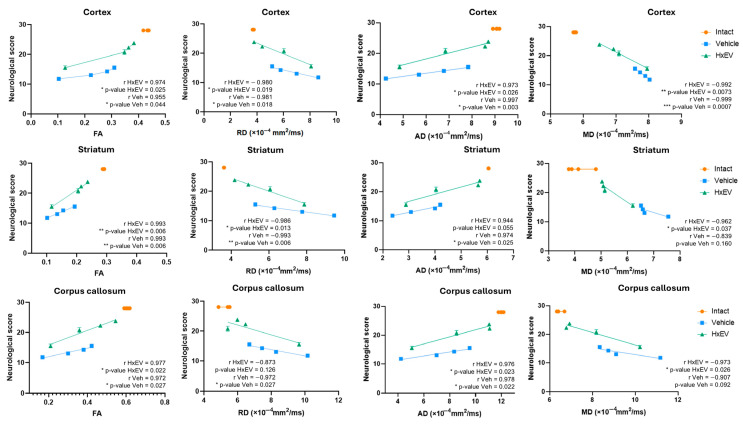
Correlation of sensorimotor performance with DTI metrics. Pearson correlation and simple linear regression were used to relate overall neurological scores to diffusion tensor biomarkers—fractional anisotropy (FA), radial diffusivity (RD), axial diffusivity (AD), and mean diffusivity (MD). Symbols denote significance: */**/*** Vehicle vs. HxEV (*p* ≤ 0.05/*p* ≤ 0.01/*p* ≤ 0.001).

In addition, correlations between global neurofunctional performance and DTI metrics (FA, RD, AD, MD) were examined to assess structure–function relationships. FA showed the strongest association with functional recovery (Figure 11).

Across the cortex, striatum, and corpus callosum, neurofunctional performance correlated strongly with FA at 1, 7, 14, and 21 days post-stroke (Pearson’s r > 0.95; R^2^ > 0.9; *p* < 0.05). Although not statistically significant, concordant trends were noted for RD in the corpus callosum (*p* = 0.12), AD in the striatum (*p* = 0.55), and MD in both the striatum (*p* = 0.16) and corpus callosum (*p* = 0.092).

The concordance between DTI parameters—particularly FA and RD—and MAP2 staining supports their translational value as non-invasive biomarkers of post-stroke structural recovery and suggests that HxEV treatment fosters a microenvironment favorable to dendritic and axonal repair in vulnerable regions.

Collectively, these data support FA and RD as the primary imaging biomarkers for monitoring post-stroke recovery and for evaluating HxEV-based interventions. Table 1 summarizes the relative utility of each DTI metric across regions and recovery phases.

## 4. Discussion

### 4.1. Reparative Potential of Hypoxia-Derived EVs in Ischemic Stroke

This study shows that extracellular vesicles (EVs) released by astrocytes under hypoxia significantly enhance post-ischemic recovery in a rodent model of cerebral infarction.

The motor cortex, striatum, and corpus callosum were selected a priori because of their structural and functional relevance to ischemic stroke. The motor cortex lies within the vascular territory of the middle cerebral artery (MCA) and is highly vulnerable to ischemia, with injury tightly linked to clinically meaningful motor deficits [16]. The striatum, supplied by MCA perforators, is a key basal ganglia structure for motor control; ischemic damage in this region causes marked functional impairment [17]. By contrast, the corpus callosum—irrigated mainly by the anterior cerebral artery—often sustains less direct ischemic injury in this model, making it informative for studying interhemispheric reorganization and microstructural repair [18]. Together, these regions provide complementary windows on lesion severity and recovery trajectories across structures with distinct vascularization and function.

We observed a progressive reduction in infarct volume from day 1 to day 21, consistent with reports that EVs of mesenchymal or neuronal origin facilitate tissue repair and functional improvement after stroke [19,20]. Although both groups showed decreasing lesion size, the decline was significantly greater with HxEV treatment, indicating preservation of structurally and functionally critical cortical and subcortical tissue and aligning with superior behavioral outcomes. Prior work supports the notion that treatment-induced differences in infarct magnitude are strong predictors of recovery [21].

The superior effects of HxEVs are plausibly mediated by delivery of neurotrophic factors (e.g., BDNF, GDNF) and by modulation of post-ischemic inflammation. Emerging evidence indicates that EV protein and RNA cargo attenuate apoptotic pathways and may reprogram endogenous cells toward neuroprotective phenotypes [22,23].

While some spontaneous recovery follows cerebral ischemia, its molecular underpinnings remain incompletely defined [24]. Here, longitudinal MRI combined with quantitative diffusion tensor imaging (DTI) provided a robust framework to track evolving structural changes and to evaluate therapeutic response over time [25].

Given the established prognostic value of infarct volume [26,27], the faster and more pronounced lesion reduction in HxEV-treated animals [28] likely represents true neuroprotection and repair. These structural gains correlated with improved motor performance, in line with earlier observations linking imaging and function [29]. Collectively, our findings support HxEVs as a promising platform to promote neuroplasticity and functional recovery after ischemic stroke.

### 4.2. Longitudinal Structural Recovery Evidenced by DTI

The sustained reduction in infarct volume from the acute phase (day 1) through the subacute and early chronic stages (day 21) indicates a prolonged neuroprotective effect of HxEVs [30]. This aligns with reports that EVs mitigate cerebral injury and promote structural and functional recovery in rodent stroke models [31,32], potentially by modulating neuroinflammation, enhancing neurogenesis, and facilitating synaptic plasticity [33,34,35].

DTI provided sensitive, quantitative biomarkers of these dynamic changes, capturing microstructural alterations beyond conventional MRI [36]. FA, MD, AD, and RD are widely accepted metrics that reflect complementary aspects of white-matter integrity with established reproducibility across regions and disease stages [37]. Reliance on a single parameter can limit interpretability—for example, FA is specific but cannot disentangle demyelination, edema, or axonal degeneration—thereby justifying a combined evaluation of FA, MD, AD, and RD to improve diagnostic and prognostic accuracy [38].

In ischemic stroke, these DTI parameters possess strong predictive value. FA and AD have been validated as biomarkers of corticospinal tract damage and correlate with motor outcomes and global recovery [39]. Moreover, axial diffusivity of the corona radiata at 24 h is a robust predictor of early motor recovery and mid-term functional status [40]. Guided by this evidence, our comparative analyses focused on FA, MD, AD, and RD to define robust biomarkers in the present study, reinforcing both the biological significance and methodological consistency of our findings.

Among these metrics, FA emerged as a particularly reliable indicator of axonal integrity, with decreases reflecting fiber disorganization and increases suggesting tract restoration [41,42]. In our cohort, progressive FA recovery in the corpus callosum—and, to a lesser extent, in cortex and striatum—indicates that HxEV treatment supports axonal preservation and remyelination. Consistent with this interpretation, the corticothalamic tract exhibited FA reductions after infarction that paralleled motor deficits in Vehicle-treated animals [43,44], whereas HxEV treatment produced a robust FA restoration over time that tracked with behavioral improvement.

RD changes further support this reparative profile: the significant RD reduction from day 7 onward in HxEV-treated rats is compatible with remyelination during the subacute stage, consistent with prior experimental demyelination studies [45]. Taken together, FA and RD were the most informative DTI indicators of tissue repair in our dataset [45,46], as shown by consistent intergroup and regional differences across the three-week follow-up.

Finally, regional patterns were not uniform. The corpus callosum showed early, sustained recovery, whereas the motor cortex demonstrated more heterogeneous trajectories—likely reflecting its complex crossing-fiber architecture [47]—a known limitation of standard DTI quantification in such regions [48].

### 4.3. Diffusion Parameters Reflect Distinct Microstructural Processes

Beyond FA, AD and RD offer complementary readouts of axonal and myelin integrity, respectively. AD indexes water motion parallel to fiber tracts, whereas RD captures diffusion perpendicular to axons; together they are sensitive to axonal injury and demyelination.

In our model, AD fell markedly across regions in the acute phase, consistent with widespread axonal disruption. HxEV-treated rats then showed progressive AD normalization from day 7, most clearly in cortex and striatum and sustained in the corpus callosum, indicating late-stage axonal repair [49,50,51,52,53,54,55,56,57].

By contrast, RD remained elevated in Vehicle animals throughout follow-up, a pattern aligned with persistent demyelination and impaired white-matter restoration [45,58,59]. HxEV administration produced significant RD reductions beginning on day 7, supporting accelerated remyelination—consistent with experimental demyelination studies in which RD normalization tracks myelin recovery [45,58,59].

MD, the average of AD and RD, was acutely increased after stroke—reflecting cytotoxic/vasogenic edema [60]—and normalized more rapidly with HxEVs. However, MD was less specific for structural repair than FA or RD, largely capturing global tissue water changes rather than finer microstructural remodeling [61,62,63].

Taken together, FA remained the most robust single biomarker of tract reorganization: HxEV-treated animals exhibited significant, sustained FA increases across regions, with the corpus callosum showing the largest recovery. This trajectory is consistent with concurrent improvements in axonal alignment and remyelination—two key processes in post-ischemic neural repair [59].

### 4.4. Regional Differences in Treatment Response

Correlating DTI metrics with functional recovery showed that FA and RD were the most reliable longitudinal biomarkers of neuroregeneration [55,64,65]. The largest effect was observed in the corpus callosum, where FA improved steadily and exceeded 50% relative to Vehicle from day 7 onward (*p* < 0.01), suggesting a particularly strong HxEV effect on densely myelinated commissural tracts. In contrast, the striatum and motor cortex showed delayed and more variable changes; significant FA and RD differences emerged in the striatum by day 14 (*p* < 0.05), whereas cortical recovery was less consistent—likely reflecting the heterogeneous organization and greater ischemic vulnerability of cortical fibers [66]. These findings are consistent with known limitations of conventional DTI in regions with extensive crossing fibers; advanced models such as HARDI or NODDI may improve sensitivity in these anatomically complex areas [56,67].

The near-linear recovery trajectory in the corpus callosum, versus the apparent saturation in cortex and striatum by week three, is biologically plausible. The callosum is largely outside the middle cerebral artery territory and thus sustains less acute ischemic damage than cortical or striatal regions. Its architecture—dominated by myelinated interhemispheric axons with comparatively low synaptic complexity—confers resilience to injury and supports sustained repair [68]. Enrichment in oligodendrocytes likely facilitates remyelination and axonal restoration, while relatively lower exposure to secondary inflammatory responses further enhances recovery efficiency [69].

Taken together, these data indicate a region-specific therapeutic impact of HxEVs: the corpus callosum shows the earliest and most robust improvement, whereas gray-matter structures (striatum and motor cortex) recover more slowly but measurably [70].

Finally, although neurotrophic factors such as BDNF and GDNF are frequently reported in astrocyte-derived exosomes under hypoxic/ischemic conditions and are implicated in neuroprotection, synaptic plasticity, and repair [71], we did not directly confirm their presence in the EVs used here. Our results nevertheless align with studies showing that neural- and mesenchymal-stem-cell–derived exosomes can deliver these cargos and improve post-stroke outcomes [72,73]. Future work should include proteomic profiling or targeted immunoblotting of EV cargo to strengthen mechanistic interpretation.

### 4.5. Correlation Between Dendro-Axonal Integrity and Histological Validation

Endogenous repair after ischemic stroke involves extensive cytoskeletal remodeling. Axonal regrowth originates at the growth cone through coordinated actin reorganization and microtubule extension, tightly regulated by extracellular cues and intracellular signaling that govern axonal sprouting and dendritic stabilization [74,75,76].

At 21 days post-tMCAO, MAP-2 immunofluorescence revealed clear structural differences between groups. Vehicle-treated rats showed a marked reduction in MAP-2-positive neurons in peri-infarct cortex, with pronounced dendritic fragmentation and disrupted axonal morphology. These changes align with DTI results—lower FA and higher RD—in the same regions, supporting the validity of DTI metrics as structural biomarkers of ischemia-induced injury. The reduction in FA and the increase in RD in Vehicle animals parallel the loss of dendritic integrity, indicating persistent microstructural disorganization and an inadequate reparative response.

Given the dendrite-rich architecture of cortex, loss of MAP-2 (a marker of dendritic degeneration) directly alters water-diffusion constraints, rendering DTI measures particularly sensitive to these histological changes [77,78]. In the striatum, MAP-2 area correlated significantly with FA but not with RD, AD, or MD, suggesting that dendritic integrity in this nucleus is more closely captured by anisotropy than by global diffusivity indices [79]. Although absolute FA values are lower in striatum because of abundant crossing fibers, the significant association with MAP-2 indicates that FA remains a sensitive indicator of dendritic alterations; RD, AD, and MD are likely more influenced by extracellular water shifts and thus less directly reflective of dendritic status [80]. Improvement in striatal FA and RD—despite the region’s gray-matter composition—further underscores the usefulness of these metrics for tracking dendritic integrity and suggests that HxEV effects extend beyond peri-infarct cortex.

Conversely, HxEV-treated rats exhibited preserved MAP-2-positive dendrites, particularly in apical dendrites within peri-infarct cortex, with more continuous axonal processes and intact morphology. These observations—improved arborization, sustained apical orientation, and preserved dendritic spines—are consistent with partial recovery of DTI parameters and support the hypothesis that HxEVs promote cytoskeletal stabilization and structural repair.

Collectively, these findings validate DTI-derived microstructural metrics as reliable readouts of underlying histological change [75,81,82] and indicate that HxEV treatment facilitates axonal regeneration and preserves neuronal architecture within ischemia-vulnerable regions, fostering a microenvironment conducive to functional recovery.

### 4.6. Regional Recovery Dynamics and Comparative Performance of DTI Biomarkers

Region-specific DTI analyses showed differential responses to ischemia and to hypoxia-conditioned astrocyte-derived EVs (HxEVs), with FA and RD emerging as the most sensitive indicators of treatment efficacy.

Corpus callosum (white matter). In Vehicle animals, FA fell by >50% at day 1, with concomitant increases in RD and MD—patterns consistent with axonal disorganization and demyelination. HxEV treatment produced an early and sustained rebound: FA improved from day 7 (*p* < 0.01), while RD and MD progressively normalized by day 21, indicating restoration of white-matter integrity through remyelination and axonal repair.

Striatum (gray matter). Given its intrinsically low anisotropy, post-stroke alterations in FA and RD were detectable from day 1. HxEVs drove partial recovery of both metrics beginning at day 14 (*p* < 0.05), consistent with preserved neuronal architecture and enhanced structural reorganization. The later onset versus white matter suggests tissue-specific repair kinetics.

Motor cortex. Changes were more heterogeneous. In Vehicle controls, MD remained elevated across the study, reflecting persistent edema and microstructural disruption. HxEVs accelerated MD normalization; however, FA and RD recovery was smaller and more variable, implying that cortical reorganization may require longer follow-up or adjunctive interventions to match subcortical gains.

Biomarker comparison. Across regions, FA reliably captured microstructural recovery in both white and gray matter, whereas RD was particularly sensitive to remyelination. MD primarily reflected acute injury and edema resolution and was less specific for later repair. AD detected axonal injury but was less discriminative for longitudinal treatment effects.

### 4.7. DTI Biomarkers as Non-Invasive Tools for Therapy Monitoring

DTI provides quantitative, non-invasive readouts of microstructural change after stroke. Each parameter captures a complementary facet of water diffusion and tissue architecture, enabling longitudinal tracking of neurorestoration. Among these metrics, FA and RD were the most sensitive to hypoxia-conditioned astrocyte-derived EV (HxEV) effects. FA—an index of axonal organization—showed early and sustained improvement in heavily myelinated commissural tracts (e.g., corpus callosum), whereas RD—sensitive to myelin integrity—declined in both cortical and subcortical regions as remyelination progressed [45,46]. FA and RD consistently distinguished HxEV-treated animals from Vehicle controls across all time points, supporting their use as primary imaging endpoints for recovery monitoring.

These observations argue for prioritizing FA and RD in preclinical and clinical studies as robust indicators of structural repair with functional relevance. Incorporating these metrics into therapeutic trials could enable real-time assessment of neuroplasticity and help optimize dosing and timing of regenerative interventions.

Overall, our data indicate that HxEVs confer meaningful neuroprotection after ischemia—preserving dendritic architecture and driving region-specific recovery. The sustained gains in the corpus callosum relative to cortex and striatum suggest that inherent microstructural and vascular features shape the tempo of repair. Together, the imaging and histological findings support a model in which astrocyte-derived EVs act not only as structural stabilizers but also as modulators of post-stroke remodeling.

### 4.8. Limitations of the Study

This work provides evidence for the neuroprotective and neurorestorative actions of hypoxia-conditioned astrocyte-derived extracellular vesicles (HxEVs), yet several limitations should be acknowledged.

First, the sample size was modest (*n* = 6 per group). Although it followed a priori power estimates, it may have reduced sensitivity to detect smaller effects in secondary outcomes and increased vulnerability to the inter-individual variability inherent to stroke models. Larger cohorts will be needed to confirm these trends and increase statistical power.

Second, follow-up was restricted to 21 days post-ischemia. This window captures acute, subacute, and early chronic phases of tissue change [81,82], coinciding with the onset of neuroplasticity, early neurovascular remodeling, and initial motor recovery [83]. Ethical considerations and alignment with the 3R principles also informed this choice; extending observation was not required to meet the study aims [84]. Nonetheless, longer studies are warranted to determine the durability of the observed benefits.

Third, the focal ischemia model used—young, otherwise healthy rats—simplifies clinical complexity and limits generalizability to patient populations with comorbidities (e.g., aging, diabetes, hypertension, atherosclerosis). Although rodent tMCAO models are widely used, they do not fully recapitulate human pathophysiology; species differences in brain physiology, immune responses, blood–brain barrier dynamics, and network organization—together with accelerated injury–recovery kinetics—can overestimate efficacy and shift optimal therapeutic windows. In addition, the molecular cargo of rodent astrocyte-derived EVs may differ from human EVs, affecting biodistribution, mechanism, and effect size. These constraints should be weighed carefully when extrapolating to clinical practice.

We intentionally employed HxEVs—EVs released by astrocytes exposed to hypoxia—because they provide a reproducible, relatively homogeneous preparation with controlled cellular origin and microenvironmental imprinting. This standardization was essential to interrogate mechanistic links between vesicle exposure and neuroprotection in vivo. We recognize that EVs isolated from biological fluids such as cerebrospinal fluid (CSF) may more closely reflect the in vivo ischemic milieu; however, their heterogeneity, inter-individual variability, and low recovery currently limit feasibility in preclinical standardization. Comparative studies directly assessing cargo and function of CSF-derived EVs will be important next steps. Prior work showing robust neuroprotection by stem-cell–derived exosomes under ischemic conditions supports the rationale for our approach [85].

Another important limitation is the absence of a comparator group treated with EVs derived from astrocytes under normoxic conditions, which would have allowed a more direct attribution of effects to hypoxic conditioning. Our primary objective, however, was to validate diffusion tensor imaging (DTI) metrics as non-invasive biomarkers of structural repair after cerebral infarction and to determine which are most specific for detecting subtle microstructural changes versus global tissue alterations. These results provide a solid basis for future, more comprehensive studies that will include a normoxic-EV control arm.

Fourth, HxEVs were delivered intracerebroventricularly. While appropriate for maximizing central exposure in proof-of-concept experiments, this route is invasive and not readily translatable. Future studies should test clinically feasible routes—intravenous, intra-arterial, or intranasal—to enhance applicability.

Obtaining human astrocyte-derived EVs for clinical use also poses substantial ethical and technical challenges. Direct procurement of primary human astrocytes is neither feasible nor ethically acceptable, and access to viable brain tissue is extremely limited. A promising alternative is to generate patient-specific astrocytes from induced pluripotent stem cells (iPSCs) derived from somatic cells (e.g., dermal fibroblasts or blood). This strategy enables personalized, immunocompatible cell sources and supports scalable, well-controlled production. However, clinical translation of iPSC-derived astrocytes still faces important hurdles: standardizing differentiation protocols to obtain homogeneous, functionally mature populations; removing undifferentiated cells with tumorigenic potential; and instituting rigorous quality-control procedures to ensure safety, genetic stability, and reproducible function. Human embryonic stem cell (hESC)–derived astrocytes are another option, but their clinical application is even more constrained by ethical, legal, and regulatory considerations in many countries.

Finally, although DTI yielded informative microstructural biomarkers, the single-tensor model per voxel is limited in regions with complex fiber architecture. Advanced diffusion methods (e.g., NODDI, HARDI) could improve specificity in these areas. Moreover, the molecular composition of the administered EVs (e.g., miRNA and protein cargo) and downstream signaling pathways were not comprehensively characterized here and merit dedicated mechanistic investigations.

### 4.9. Future Perspectives and Potential Applications

While our findings support the biological activity of HxEVs in a rat tMCAO model, translation to humans must proceed cautiously and only after a staged preclinical program is satisfied.

Near-term preclinical priorities include independent replication; dose–response and therapeutic-window studies; longer follow-up; pharmacokinetic/biodistribution profiling (brain exposure, persistence, off-target accumulation); repeated-dose and late-toxicity assessments in aged and comorbidity-bearing models; and head-to-head comparisons with normoxia-derived EVs and standard-of-care therapies.

Manufacturing and quality efforts should establish GMP-compatible production of human-source astrocyte EVs (e.g., iPSC-derived) with validated identity, purity, potency, batch consistency, and stability assays, and with analytics that bridge rodent HxEV cargo to human products.

Clinical practicality requires abandoning intracerebroventricular delivery in favor of comparative evaluation of clinically realistic routes (intravenous, intra-arterial, intranasal) for brain target engagement and safety, rather than assuming that lower invasiveness alone ensures efficacy.

Endpoints and decision gates. If these criteria are met, early translational studies could use FA and RD as exploratory pharmacodynamic markers of white-matter integrity—complementing clinical and functional outcomes, and acknowledging known DTI limitations. Where appropriate, advanced diffusion models (e.g., NODDI, HARDI) and histological/proteomic readouts should be incorporated to strengthen mechanistic inference.

In sum, the present results are hypothesis-generating and provide a methodological foundation for a cautious, data-driven translational path. Meeting the preclinical, manufacturing, delivery, and endpoint standards outlined above will determine whether HxEVs can justifiably advance toward early-phase human investigation [86].

### 4.10. Relevance of Findings

The present work advances both mechanistic understanding and translational planning for EV-based interventions after cerebral ischemia. First, the quantitative, longitudinal design—combining MRI-derived DTI metrics with histological validation—provides objective evidence for spontaneous and ADEV-modulated recovery processes, reducing reliance on indirect behavioral readouts [87].

Second, a multimodal analytic approach showed that no single metric captures all stages of repair; rather, hierarchies of sensitivity emerge across time, with FA and RD outperforming AD and MD for detecting microstructural change during subacute recovery [88].

Third, the data support the concept that post-stroke repair is temporally stratified, comprising overlapping phases of edema resolution, axonal reorganization, remyelination, and synaptic remodeling [89]. The ability of hypoxia-conditioned ADEVs to influence these processes is consistent with prior work implicating neurotrophic and microRNA cargo in plasticity-related pathways and immune modulation [90]. This supports EV-based strategies as credible tools to augment endogenous repair.

Finally, identifying FA and RD as sensitive, non-invasive biomarkers strengthens the feasibility of incorporating imaging endpoints into future preclinical and clinical studies, enabling more objective monitoring and optimization of EV-based therapies for ischemic stroke [91].

## 5. Conclusions

DTI-derived biomarkers provide a sensitive, non-invasive window on dynamic structural changes during post-ischemic recovery. Among them, fractional anisotropy (FA) and radial diffusivity (RD) were the most informative indicators of axonal organization and myelin integrity across regions.

Intracerebroventricular delivery of hypoxia-conditioned astrocyte-derived EVs (HxEVs) was associated with early and sustained reductions in infarct volume, accelerated normalization of diffusion parameters, and improved neurological outcomes in a rat tMCAO model. Benefits were most pronounced in white-matter tracts—particularly the corpus callosum—while gray-matter regions showed heterogeneous but meaningful improvement. Histology corroborated imaging findings, with preservation of MAP-2–positive dendritic architecture in ADEV-treated animals.

These results highlight region-specific recovery dynamics and support FA and RD as primary imaging biomarkers for therapy monitoring. Collectively, the data provide strong preclinical evidence that HxEVs foster a reparative microenvironment after stroke. Future work should refine dose and timing, evaluate clinically feasible delivery routes, and verify EV molecular cargo with proteomic assays, while validating these markers in larger and more clinically relevant models.

## Figures and Tables

**Table 1:** Comparative effectiveness of DTI biomarkers by region and time.

Biomarker	Global Effectiveness	Most Responsive Region	Peak Differential Time	Intergroup Significance
FA	High	Corpus callosum	Day 7	<0.0001
RD	High	Striatum	Days 7–14	<0.01
AD	Moderate	Cortex	Day 21	<0.05
MD	Low	Cortex	Day 1	NS

## Data Availability

Information is available upon reasonable request to the corresponding author.

## Abbreviations

The following abbreviations are used in this manuscript:

ADEV	astrocyte-derived extracellular vesicle
HxEV	hypoxia-conditioned extracellular vesicle
EV	extracellular vesicle
DTI	diffusion tensor imaging
MRI	magnetic resonance imaging
FA	fractional anisotropy
RD	radial diffusivity
AD	axial diffusivity
MD	mean diffusivity
tMCAO	transient middle cerebral artery occlusion
MCAO	middle cerebral artery occlusion
MCA	middle cerebral artery
CCA	common carotid artery
ICA	internal carotid artery
ECA	external carotid artery
CBF	cerebral blood flow
i.c.v.	intracerebroventricular
FSEMS	fast spin echo multislice
ROI	region of interest
GFAP	glial fibrillary acidic protein
PFA	paraformaldehyde
BSA	bovine serum albumin
DAPI	4′,6-diamidino-2-phenylindole
PBS	phosphate-buffered saline
TEM	transmission electron microscopy
MRS	magnetic resonance spectroscopy
NODDI	neurite orientation dispersion and density imaging
HARDI	high-angular-resolution diffusion imaging
mNSS	modified neurological severity score
CSF	cerebrospinal fluid
SEM	standard error of the mean
TE	echo time
TR	repetition time

## Appendix A

**Table A1:** Neurofunctional scoring system used to evaluate sensorimotor recovery following ischemic stroke in rats. A 10-item composite scale was used to assess neurological deficits and spontaneous recovery following middle cerebral artery occlusion (tMCAO) in rats. Each domain evaluates motor (M), sensory (S), or sensorimotor (SM) function. Scores range from 0 (severe deficit) to 3 (normal performance), with higher total scores indicating better neurofunctional status. This battery was applied longitudinally at 1, 7, 14, and 21 days post-stroke by a blinded evaluator. The scale integrates spontaneous activity, gait coordination, postural responses, vibrissae stimulation, and proprioceptive integrity.

Domain	Functional Category	Scoring Criteria (0–3)	Max Score
Spontaneous Activity	Sensorimotor	0: No exploration 1: Explores <10 sec 2: Explores 10–20 sec 3: Explores >20 sec	3
Gait Coordination	Motor	0: No support 1: Drags dorsum 2: Supports ulnar side 3: Symmetrical movement	3
Forelimb Grasp	Motor	0: Falls 1: Cannot grasp 2: Asymmetric grip 3: Symmetrical grip	3
Suspension Response	Motor	0: No response 1: Asymmetrical lift 2: Symmetrical lift	3
Circling Behavior	Motor	0: No movement 1: Stimulus-induced circles 2: Sporadic spontaneous circles 3: Constant spontaneous circling	3
Cylinder Test	Sensorimotor	0: No support 1: Unequal forelimb use 2: Equal forelimb support	3
Body Posture	Sensorimotor	0: Cannot maintain posture 1: Body tilt 2: Head tilt with forelimb extension 3: Balanced posture	3
Forelimb Retraction	Sensorimotor	0: No flexion 1: Slight ipsilateral movement 2: Asymmetric retraction 3: Symmetric retraction and displacement	3
Vibrissae Stimulation	Sensory	0: No response bilaterally 1: Absent contralateral response 2: Moderate contralateral response 3: Symmetric vibrissae response	3
Body Proprioception	Sensory	0: No bilateral response 1: No response on affected side 2: No thoracic response 3: Symmetrical response to bilateral tactile stimulation	3

## References

[1] Liu S., Li Y., Lan X., Wang L., Li H., Gu D., Wang M., Liu J. (2025). Global, regional, and national trends in ischaemic stroke burden and risk factors among adults aged 20 + years (1990–2021): A systematic analysis of data from the global burden of disease study 2021 with projections into 2050. Front. Public Health.

[2] Saini V., Guada L., Yavagal D.R. (2021). Global epidemiology of stroke and access to acute ischemic stroke interventions. Neurology.

[3] Gualerzi A., Picciolini S., Roda F., Bedoni M. (2021). Extracellular vesicles in regeneration and rehabilitation recovery after stroke. Biology.

[4] Mosquera-Heredia M.I., Morales L.C., Vidal O.M., Barcelo E., Silvera-Redondo C., Velez J.I., Garavito-Galofre P. (2021). Exosomes: Potential disease biomarkers and new therapeutic targets. Biomedicines.

[5] Jung H., Jung Y., Seo J., Bae Y., Kim H.S., Jeong W. (2024). Roles of extracellular vesicles from mesenchymal stem cells in regeneration. Mol. Cells.

[6] Wang X., Li A., Fan H., Li Y., Yang N., Tang Y. (2024). Astrocyte-derived extracellular vesicles for ischemic stroke: Therapeutic potential and prospective. Aging Dis..

[7] Mukerjee N., Bhattacharya A., Maitra S., Kaur M., Ganesan S., Mishra S., Ashraf A., Rizwan M., Kesari K.K., Tabish T.A. (2025). Exosome isolation and characterization for advanced diagnostic and therapeutic applications. Mater. Today Bio.

[8] Mondragon-Lozano R., Diaz-Ruiz A., Rios C., Olayo Gonzalez R., Favila R., Salgado-Ceballos H., Roldan-Valadez E. (2013). Feasibility of in vivo quantitative magnetic resonance imaging with diffusion weighted imaging, t2-weighted relaxometry, and diffusion tensor imaging in a clinical 3 tesla magnetic resonance scanner for the acute traumatic spinal cord injury of rats: Technical note. Spine.

[9] Percie du Sert N., Hurst V., Ahluwalia A., Alam S., Avey M.T., Baker M., Browne W.J., Clark A., Cuthill I.C., Dirnagl U. (2020). The arrive guidelines 2.0: Updated guidelines for reporting animal research. J. Cereb. Blood Flow. Metab..

[10] Curtin L.I., Grakowsky J.A., Suarez M., Thompson A.C., DiPirro J.M., Martin L.B., Kristal M.B. (2009). Evaluation of buprenorphine in a postoperative pain model in rats. Comp. Med..

[11] Dickens A.M., Tovar Y.R.L.B., Yoo S.W., Trout A.L., Bae M., Kanmogne M., Megra B., Williams D.W., Witwer K.W., Gacias M. (2017). Astrocyte-shed extracellular vesicles regulate the peripheral leukocyte response to inflammatory brain lesions. Sci. Signal..

[12] Burguete M.C., Torregrosa G., Perez-Asensio F.J., Castello-Ruiz M., Salom J.B., Gil J.V., Alborch E. (2006). Dietary phytoestrogens improve stroke outcome after transient focal cerebral ischemia in rats. Eur. J. Neurosci..

[13] Zhang H., Shen Y., Wang W., Gao H. (2015). Rat model of focal cerebral ischemia in the dominant hemisphere. Int. J. Clin. Exp. Med..

[14] Oh S.S., Narver H.L. (2024). Mouse and rat anesthesia and analgesia. Curr. Protoc..

[15] Cortez-Conradis D., Favila R., Isaac-Olive K., Martinez-Lopez M., Rios C., Roldan-Valadez E. (2013). Diagnostic performance of regional dti-derived tensor metrics in glioblastoma multiforme: Simultaneous evaluation of p, q, l, cl, cp, cs, ra, rd, ad, mean diffusivity and fractional anisotropy. Eur. Radiol..

[16] Scheulin K.M., Jurgielewicz B.J., Spellicy S.E., Waters E.S., Baker E.W., Kinder H.A., Simchick G.A., Sneed S.E., Grimes J.A., Zhao Q. (2021). Exploring the predictive value of lesion topology on motor function outcomes in a porcine ischemic stroke model. Sci. Rep..

[17] de la Rosa-Prieto C., Laterza C., Gonzalez-Ramos A., Wattananit S., Ge R., Lindvall O., Tornero D., Kokaia Z. (2017). Stroke alters behavior of human skin-derived neural progenitors after transplantation adjacent to neurogenic area in rat brain. Stem Cell Res. Ther..

[18] Li Y., Wu P., Liang F., Huang W. (2015). The microstructural status of the corpus callosum is associated with the degree of motor function and neurological deficit in stroke patients. PLoS ONE.

[19] Xin H., Li Y., Cui Y., Yang J.J., Zhang Z.G., Chopp M. (2013). Systemic administration of exosomes released from mesenchymal stromal cells promote functional recovery and neurovascular plasticity after stroke in rats. J. Cereb. Blood Flow Metab..

[20] Doeppner T.R., Kaltwasser B., Bahr M., Hermann D.M. (2014). Effects of neural progenitor cells on post-stroke neurological impairment-a detailed and comprehensive analysis of behavioral tests. Front. Cell Neurosci..

[21] Cherian S.G., Narayan S.K., Arumugam M. (2023). Exosome therapies improve outcome in rodents with ischemic stroke; meta-analysis. Brain Res..

[22] Zhang Y., Liu Y., Liu H., Tang W.H. (2019). Exosomes: Biogenesis, biologic function and clinical potential. Cell Biosci..

[23] Broughton B.R., Reutens D.C., Sobey C.G. (2009). Apoptotic mechanisms after cerebral ischemia. Stroke.

[24] Azizi F., Askari S., Javadpour P., Hadjighassem M., Ghasemi R. (2020). Potential role of exosome in post-stroke reorganization and/or neurodegeneration. EXCLI J..

[25] Arnberg F., Lundberg J., Soderman M., Damberg P., Holmin S. (2012). Image-guided method in the rat for inducing cortical or striatal infarction and for controlling cerebral blood flow under mri. Stroke.

[26] Liu F., Yao Y., Zhu B., Yu Y., Ren R., Hu Y. (2024). The novel imaging methods in diagnosis and assessment of cerebrovascular diseases: An overview. Front. Med..

[27] Milidonis X., Marshall I., Macleod M.R., Sena E.S. (2015). Magnetic resonance imaging in experimental stroke and comparison with histology: Systematic review and meta-analysis. Stroke.

[28] Zaidi S.F., Aghaebrahim A., Urra X., Jumaa M.A., Jankowitz B., Hammer M., Nogueira R., Horowitz M., Reddy V., Jovin T.G. (2012). Final infarct volume is a stronger predictor of outcome than recanalization in patients with proximal middle cerebral artery occlusion treated with endovascular therapy. Stroke.

[29] Gaudinski M.R., Henning E.C., Miracle A., Luby M., Warach S., Latour L.L. (2008). Establishing final infarct volume: Stroke lesion evolution past 30 days is insignificant. Stroke.

[30] Wang Y., Li M., Jiang Y., Ji Q. (2024). Comparative efficacy of neuroprotective agents for improving neurological function and prognosis in acute ischemic stroke: A network meta-analysis. Front. Neurosci..

[31] Ouro A., Rodriguez-Diaz A., Lopez-Gonzalez T., Romaus-Sanjurjo D., Estevez-Salguero A., Iglesias-Rey R., Rodriguez-Arrizabalaga M., Fernandez-Sanmartin P., Castro-Mosquera M., Debasa-Mouce M. (2025). Neuroprotective effect of small extracellular vesicle-mediated targeting of ampkalpha2 in cerebral ischemia. Metabolism.

[32] Zhang H.X., Tao L.Q., Chen Y.H., Jiang T.Y., Ye Z.Y., She W., Chen C.Y., Han Y.L., Qi C., Shen C. (2025). Enhanced therapeutic effects of extracellular vesicles targeting mir-137 contribute to functional recovery by attenuating neuronal injury after ischemic stroke. Neurosci. Bull..

[33] Ahmed W., Kuniyan M.S., Jawed A.M., Chen L. (2023). Engineered extracellular vesicles for drug delivery in therapy of stroke. Pharmaceutics.

[34] Hermann D.M., Peruzzotti-Jametti L., Giebel B., Pluchino S. (2024). Extracellular vesicles set the stage for brain plasticity and recovery by multimodal signalling. Brain.

[35] Kumar M.A., Baba S.K., Sadida H.Q., Marzooqi S.A., Jerobin J., Altemani F.H., Algehainy N., Alanazi M.A., Abou-Samra A.B., Kumar R. (2024). Extracellular vesicles as tools and targets in therapy for diseases. Signal Transduct. Target. Ther..

[36] Choudhri A.F., Chin E.M., Blitz A.M., Gandhi D. (2014). Diffusion tensor imaging of cerebral white matter: Technique, anatomy, and pathologic patterns. Radiol. Clin. North. Am..

[37] Lepomaki V.K., Paavilainen T.P., Hurme S.A., Komu M.E., Parkkola R.K., group P.s. (2012). Fractional anisotropy and mean diffusivity parameters of the brain white matter tracts in preterm infants: Reproducibility of region-of-interest measurements. Pediatr. Radiol..

[38] Mendez Colmenares A., Hefner M.B., Calhoun V.D., Salerno E.A., Fanning J., Gothe N.P., McAuley E., Kramer A.F., Burzynska A.Z. (2023). Symmetric data-driven fusion of diffusion tensor mri: Age differences in white matter. Front. Neurol..

[39] Doughty C., Wang J., Feng W., Hackney D., Pani E., Schlaug G. (2016). Detection and predictive value of fractional anisotropy changes of the corticospinal tract in the acute phase of a stroke. Stroke.

[40] Moulton E., Amor-Sahli M., Perlbarg V., Pires C., Crozier S., Galanaud D., Valabregue R., Yger M., Baronnet-Chauvet F., Samson Y. (2015). Axial diffusivity of the corona radiata at 24 hours post-stroke: A new biomarker for motor and global outcome. PLoS ONE.

[41] Oida T., Nagahara S., Kobayashi T. (2011). Acquisition parameters for diffusion tensor imaging to emphasize fractional anisotropy: Phantom study. Magn. Reson. Med. Sci..

[42] Lipton M.L., Kim N., Park Y.K., Hulkower M.B., Gardin T.M., Shifteh K., Kim M., Zimmerman M.E., Lipton R.B., Branch C.A. (2012). Robust detection of traumatic axonal injury in individual mild traumatic brain injury patients: Intersubject variation, change over time and bidirectional changes in anisotropy. Brain Imaging Behav..

[43] Rossi-Pool R., Zainos A., Alvarez M., Diaz-deLeon G., Romo R. (2021). A continuum of invariant sensory and behavioral-context perceptual coding in secondary somatosensory cortex. Nat. Commun..

[44] Bauer A.Q., Kraft A.W., Wright P.W., Snyder A.Z., Lee J.M., Culver J.P. (2014). Optical imaging of disrupted functional connectivity following ischemic stroke in mice. Neuroimage.

[45] Song S.K., Sun S.W., Ju W.K., Lin S.J., Cross A.H., Neufeld A.H. (2003). Diffusion tensor imaging detects and differentiates axon and myelin degeneration in mouse optic nerve after retinal ischemia. Neuroimage.

[46] Beaulieu C. (2002). The basis of anisotropic water diffusion in the nervous system—A technical review. NMR Biomed..

[47] Tuch D.S., Reese T.G., Wiegell M.R., Makris N., Belliveau J.W., Wedeen V.J. (2002). High angular resolution diffusion imaging reveals intravoxel white matter fiber heterogeneity. Magn. Reson. Med..

[48] Zhang H., Schneider T., Wheeler-Kingshott C.A., Alexander D.C. (2012). Noddi: Practical in vivo neurite orientation dispersion and density imaging of the human brain. Neuroimage.

[49] Westin C.F., Maier S.E., Mamata H., Nabavi A., Jolesz F.A., Kikinis R. (2002). Processing and visualization for diffusion tensor mri. Med. Image Anal..

[50] Ranzenberger L.R., Snyder T. (2021). Diffusion Tensor Imaging.

[51] Pinter D., Gattringer T., Fandler-Hofler S., Kneihsl M., Eppinger S., Deutschmann H., Pichler A., Poltrum B., Reishofer G., Ropele S. (2020). Early progressive changes in white matter integrity are associated with stroke recovery. Transl. Stroke Res..

[52] Roldan-Valadez E., Rios-Piedra E., Favila R., Alcauter S., Rios C. (2012). Diffusion tensor imaging-derived measures of fractional anisotropy across the pyramidal tract are influenced by the cerebral hemisphere but not by gender in young healthy volunteers: A split-plot factorial analysis of variance. Chin. Med. J..

[53] Flores-Alvarez E., Durand-Munoz C., Cortes-Hernandez F., Munoz-Hernandez O., Moreno-Jimenez S., Roldan-Valadez E. (2019). Clinical significance of fractional anisotropy measured in peritumoral edema as a biomarker of overall survival in glioblastoma: Evidence using correspondence analysis. Neurol. India.

[54] Roldan-Valadez E., Anaya-Sanchez S., Rivera-Sotelo N., Moreno-Jimenez S. (2022). Diffusion tensor imaging-derived biomarkers performance in glioblastoma tumor regions: Exploratory data analysis using zombie plots and diagnostic tests. Gac. Med. Mex..

[55] Aung W.Y., Mar S., Benzinger T.L. (2013). Diffusion tensor mri as a biomarker in axonal and myelin damage. Imaging Med..

[56] Fan Q., Tian Q., Ohringer N.A., Nummenmaa A., Witzel T., Tobyne S.M., Klawiter E.C., Mekkaoui C., Rosen B.R., Wald L.L. (2019). Age-related alterations in axonal microstructure in the corpus callosum measured by high-gradient diffusion mri. Neuroimage.

[57] Hinman J.D. (2014). The back and forth of axonal injury and repair after stroke. Curr. Opin. Neurol..

[58] Cisneros-Mejorado A.J., Garay E., Ortiz-Retana J., Concha L., Moctezuma J.P., Romero S., Arellano R.O. (2020). Demyelination-remyelination of the rat caudal cerebellar peduncle evaluated with magnetic resonance imaging. Neuroscience.

[59] Becerra-Laparra I., Cortez-Conradis D., Garcia-Lazaro H.G., Martinez-Lopez M., Roldan-Valadez E. (2020). Radial diffusivity is the best global biomarker able to discriminate healthy elders, mild cognitive impairment, and alzheimer’s disease: A diagnostic study of dti-derived data. Neurol. India.

[60] DiBella E.V.R., Sharma A., Richards L., Prabhakaran V., Majersik J.J., HashemizadehKolowri S.K. (2022). Beyond diffusion tensor mri methods for improved characterization of the brain after ischemic stroke: A review. AJNR Am. J. Neuroradiol..

[61] Ellingson B.M., Mayer E., Harris R.J., Ashe-McNally C., Naliboff B.D., Labus J.S., Tillisch K. (2013). Diffusion tensor imaging detects microstructural reorganization in the brain associated with chronic irritable bowel syndrome. Pain.

[62] Baron C.A., Kate M., Gioia L., Butcher K., Emery D., Budde M., Beaulieu C. (2015). Reduction of diffusion-weighted imaging contrast of acute ischemic stroke at short diffusion times. Stroke.

[63] Pasco A., Ter Minassian A., Chapon C., Lemaire L., Franconi F., Darabi D., Caron C., Benoit J.P., Le Jeune J.J. (2006). Dynamics of cerebral edema and the apparent diffusion coefficient of water changes in patients with severe traumatic brain injury. A prospective mri study. Eur. Radiol..

[64] Hutchinson E.B., Schwerin S.C., Avram A.V., Juliano S.L., Pierpaoli C. (2018). Diffusion mri and the detection of alterations following traumatic brain injury. J. Neurosci. Res..

[65] Wang L.E., Tittgemeyer M., Imperati D., Diekhoff S., Ameli M., Fink G.R., Grefkes C. (2012). Degeneration of corpus callosum and recovery of motor function after stroke: A multimodal magnetic resonance imaging study. Hum. Brain Mapp..

[66] Weber R.A., Hui E.S., Jensen J.H., Nie X., Falangola M.F., Helpern J.A., Adkins D.L. (2015). Diffusional kurtosis and diffusion tensor imaging reveal different time-sensitive stroke-induced microstructural changes. Stroke.

[67] Palacios E.M., Owen J.P., Yuh E.L., Wang M.B., Vassar M.J., Ferguson A.R., Diaz-Arrastia R., Giacino J.T., Okonkwo D.O., Robertson C.S. (2020). The evolution of white matter microstructural changes after mild traumatic brain injury: A longitudinal dti and noddi study. Sci. Adv..

[68] Barbaresi P., Fabri M., Lorenzi T., Sagrati A., Morroni M. (2024). Intrinsic organization of the corpus callosum. Front. Physiol..

[69] Huang Z., Zhang Y., Zou P., Zong X., Zhang Q. (2025). Myelin dysfunction in aging and brain disorders: Mechanisms and therapeutic opportunities. Mol. Neurodegener..

[70] Babaeeghazvini P., Rueda-Delgado L.M., Gooijers J., Swinnen S.P., Daffertshofer A. (2021). Brain structural and functional connectivity: A review of combined works of diffusion magnetic resonance imaging and electro-encephalography. Front. Hum. Neurosci..

[71] de Rezende V.L., Mathias K., de Aguiar da Costa M., Goncalves C.L., Barichello T., Petronilho F. (2025). The role of extracellular vesicles in brain-peripheral communication following ischemic stroke: Implications for neural repair and regeneration. J. Neurochem..

[72] Zhu Z.H., Jia F., Ahmed W., Zhang G.L., Wang H., Lin C.Q., Chen W.H., Chen L.K. (2023). Neural stem cell-derived exosome as a nano-sized carrier for bdnf delivery to a rat model of ischemic stroke. Neural Regen. Res..

[73] Waseem A., Saudamini, Haque R., Janowski M., Raza S.S. (2023). Mesenchymal stem cell-derived exosomes: Shaping the next era of stroke treatment. Neuroprotection.

[74] Da Silva J.S., Medina M., Zuliani C., Di Nardo A., Witke W., Dotti C.G. (2003). Rhoa/rock regulation of neuritogenesis via profilin iia-mediated control of actin stability. J. Cell Biol..

[75] Bradke F., Fawcett J.W., Spira M.E. (2012). Assembly of a new growth cone after axotomy: The precursor to axon regeneration. Nat. Rev. Neurosci..

[76] Robinson R.A., Griffiths S.C., van de Haar L.L., Malinauskas T., van Battum E.Y., Zelina P., Schwab R.A., Karia D., Malinauskaite L., Brignani S. (2021). Simultaneous binding of guidance cues net1 and rgm blocks extracellular neo1 signaling. Cell.

[77] Mages B., Fuhs T., Aleithe S., Blietz A., Hobusch C., Hartig W., Schob S., Krueger M., Michalski D. (2021). The cytoskeletal elements map2 and nf-l show substantial alterations in different stroke models while elevated serum levels highlight especially map2 as a sensitive biomarker in stroke patients. Mol. Neurobiol..

[78] Umesh Rudrapatna S., Wieloch T., Beirup K., Ruscher K., Mol W., Yanev P., Leemans A., van der Toorn A., Dijkhuizen R.M. (2014). Can diffusion kurtosis imaging improve the sensitivity and specificity of detecting microstructural alterations in brain tissue chronically after experimental stroke? Comparisons with diffusion tensor imaging and histology. Neuroimage.

[79] Jiang Q., Zhang Z.G., Ding G.L., Silver B., Zhang L., Meng H., Lu M., Pourabdillah-Nejed D.S., Wang L., Savant-Bhonsale S. (2006). Mri detects white matter reorganization after neural progenitor cell treatment of stroke. Neuroimage.

[80] Winklewski P.J., Sabisz A., Naumczyk P., Jodzio K., Szurowska E., Szarmach A. (2018). Understanding the physiopathology behind axial and radial diffusivity changes-what do we know?. Front. Neurol..

[81] Rehman S., Nadeem A., Akram U., Sarwar A., Quraishi A., Siddiqui H., Malik M.A.J., Nabi M., Ul Haq I., Cho A. (2024). Molecular mechanisms of ischemic stroke: A review integrating clinical imaging and therapeutic perspectives. Biomedicines.

[82] Cirillo C., Brihmat N., Castel-Lacanal E., Le Friec A., Barbieux-Guillot M., Raposo N., Pariente J., Viguier A., Simonetta-Moreau M., Albucher J.F. (2020). Post-stroke remodeling processes in animal models and humans. J. Cereb. Blood Flow Metab..

[83] Lake E.M.R., Bazzigaluppi P., Mester J., Thomason L.A.M., Janik R., Brown M., McLaurin J., Carlen P.L., Corbett D., Stanisz G.J. (2017). Neurovascular unit remodelling in the subacute stage of stroke recovery. Neuroimage.

[84] Percie du Sert N., Alfieri A., Allan S.M., Carswell H.V., Deuchar G.A., Farr T.D., Flecknell P., Gallagher L., Gibson C.L., Haley M.J. (2017). The improve guidelines (ischaemia models: Procedural refinements of in vivo experiments). J. Cereb. Blood Flow. Metab..

[85] Sun X., Jung J.H., Arvola O., Santoso M.R., Giffard R.G., Yang P.C., Stary C.M. (2019). Stem cell-derived exosomes protect astrocyte cultures from in vitro ischemia and decrease injury as post-stroke intravenous therapy. Front. Cell. Neurosci..

[86] Flores-Alvarez E., Anselmo Rios Piedra E., Cruz-Priego G.A., Durand-Munoz C., Moreno-Jimenez S., Roldan-Valadez E. (2020). Correlations between dti-derived metrics and mrs metabolites in tumour regions of glioblastoma: A pilot study. Radiol. Oncol..

[87] Bang O.Y., Kim E.H., Cho Y.H., Oh M.J., Chung J.W., Chang W.H., Kim Y.H., Yang S.W., Chopp M. (2022). Circulating extracellular vesicles in stroke patients treated with mesenchymal stem cells: A biomarker analysis of a randomized trial. Stroke.

[88] Otero-Ortega L., Laso-Garcia F., Gomez-de Frutos M.D., Rodriguez-Frutos B., Pascual-Guerra J., Fuentes B., Diez-Tejedor E., Gutierrez-Fernandez M. (2017). White matter repair after extracellular vesicles administration in an experimental animal model of subcortical stroke. Sci. Rep..

[89] Heras-Romero Y., Morales-Guadarrama A., Santana-Martinez R., Ponce I., Rincon-Heredia R., Poot-Hernandez A.C., Martinez-Moreno A., Urrieta E., Bernal-Vicente B.N., Campero-Romero A.N. (2022). Improved post-stroke spontaneous recovery by astrocytic extracellular vesicles. Mol. Ther..

[90] Bernal Vicente B.N., Ponce I., Santos Gutierrez M., Rios Castro E., Tovar Y.R.L.B. (2025). Neuroprotective proteins in hypoxia-stressed astrocyte-derived extracellular vesicles. Curr. Neuropharmacol..

[91] Li Y., Tang Y., Yang G.Y. (2021). Therapeutic application of exosomes in ischaemic stroke. Stroke Vasc. Neurol..

